# Effect of Preoperative Atrial Fibrillation on Postoperative Outcome following Cardiac Surgery

**DOI:** 10.1155/2012/272384

**Published:** 2012-07-30

**Authors:** Nael Al-Sarraf, Lukman Thalib, Anne Hughes, Michael Tolan, Vincent Young, Eillish McGovern

**Affiliations:** ^1^Department of Cardiothoracic Surgery, St. James's Hospital, Dublin 8, Ireland; ^2^Department of Cardiothoracic Surgery, Chest Disease Hospital, Al-Jabriah, P.O. Box 718, Kuwait City 46308, Kuwait; ^3^Department of Community Medicine (Biostatistics), Kuwait University, Safat 13110, Kuwait

## Abstract

Atrial fibrillation remains the commonest arrhythmia encountered in cardiac surgery. Data on the effect of preoperative atrial fibrillation on postoperative outcome remain limited. We sought to assess the effects preoperative atrial fibrillation on patients' outcome following cardiac surgery. This is a retrospective review of prospectively collected departmental data of all patients who underwent cardiac surgery over 8-year period. Our cohort consisted of 3777 consecutive patients divided into atrial fibrillation (*n* = 413, 11%) and sinus rhythm (*n* = 3364, 89%). Postoperative complications and in-hospital mortality were analysed. Univariate analysis showed significantly increased mortality and major complications in atrial fibrillation compared to sinus rhythm patients. Using multiple logistic regression analysis and after accounting for Euro SCORE as a confounding variable, we found that preoperative atrial fibrillation significantly increases the risk of mortality (OR 1.7), low cardiac output state (OR 1.3), prolonged ventilation (OR 1.4), infective complication (OR 1.5), gastrointestinal complications (OR 2.0), and intensive care unit readmission (OR 1.6). Preoperative atrial fibrillation in cardiac surgery patients increases their risk of mortality and major complications following cardiac surgery. Surgical strategies such as Cox-Maze procedure may be beneficial in these patients.

## 1. Introduction

Atrial fibrillation (A Fib) is the most common arrhythmia seen in cardiac surgery. In addition, it has also been identified in nonsurgical patients as a marker of severe cardiac disease and a risk factor for decreased long-term survival [1]. Advancing age has been shown to have a significant association with the incidence of A Fib, a relationship that is particularly important as the number of elderly patients referred for surgical revascularization is increasing [2]. Data from the society of thoracic surgery national adults cardiac database collected during 2002 and 2003 revealed that the prevalence of A Fib was 5.3% among patients undergoing isolated coronary artery bypass graft (CABG) but it increased to 6.1% in 2005 [3]. Although previous studies have examined the effects of preoperative A Fib on mortality following cardiac surgery, the full spectrum of postoperative complications that might be encountered in such patients has not been reported. In addition, previous studies have concentrated on one type of cardiac surgery than the full spectrum of surgeries which might have impacted on the outcomes observed (e.g., CABG only, or aortic valve replacements alone or mitral surgeries only). We sought to assess the effect of preoperative A Fib on postoperative outcome in patients undergoing cardiac surgery in order to understand the potential deleterious effects of atrial fibrillation.

## 2. Patients and Methods

### 2.1. Patients

This is a retrospective review of a prospective database (Patient Analysis and Tracking System, Dendrite Clinical, UK). All patients who underwent isolated CABG, valve surgery, or combination of both at St James's hospital between January 2000 and July 2008 were reviewed. Three thousands and seven hundreds and seventy seven (*n* = 3777) consecutive patients were included in the study. Patients were divided into two groups based on their preoperative rhythm status: A Fib (*n* = 413, 11%) and normal sinus rhythm (*n* = 3364, 89%). Patients with other types of arrhythmias or those with automated implantable cardioverter defibrillator (AICD) were excluded from the study. All data were collected prospectively in a departmental database through the input of a dedicated database manager. The database was strictly controlled with twice yearly departmental reports generated and an annual report of departmental performance generated for audit purposes. All missing information was collected by reviewing patients' charts. Outcome measures studied were all types of postoperative complications, in-hospital mortality and length of both hospital and intensive care unit (ICU) stay. This study and the database were both approved by our institutional review board. Individual patient consent was obtained for entry into the database. However, our institutional review board waived the need for individual patient consent for this study. 

### 2.2. Definitions

All CABGs were performed through a median sternotomy. Operative priority was determined by cardiothoracic surgeons according to standard criteria. Emergency/salvage surgery refers to medical factors relating to patient's cardiac disease dictating that surgery should be performed within hours to prevent morbidity or death. Urgent operation means that medical factors require the patient to have an operation during the same admission (i.e., before discharge). Elective operation means that medical factors indicate the need for an operation through a readmission at a later date. Renal complications refer to postoperative renal failure that required dialysis or managed conservatively in patients with no prior history of same or patients with preexisting renal impairment that worsened after the surgery requiring dialysis. Neurologic complications refer to the incidence of transient ischemic attacks or permanent strokes. Gastrointestinal complications refer to gastrointestinal bleed, pancreatitis, as well as bowel ischemia and obstruction. Infective complications refer to sternal/leg wound infections (requiring antibiotics or surgical intervention) and sepsis. Pulmonary complications refer to postoperative chest infection, tracheostomy insertion, pleural effusion requiring drainage, acute respiratory distress syndrome, respiratory arrest, and reintubation. Blood transfusion requirement refers to the need of transfusion of packed red blood cells and excluding the need of isolated platelet or fresh frozen plasma transfusion.

### 2.3. Data Analysis

Data analysis began by exploring the differences between the two groups for clinical, admission, and outcome variables. Categorical variables were compared using the *Z* test for proportion or Fisher's exact test as appropriate. Continuous variables were compared using independent *t*-test or the nonparametric Mann-Whiteney test based on the satisfaction of the normality assumption (Tables 1 and 2). The effect of atrial fibrillation on mortality and other patient outcome variables was further analyzed using logistic regression methods. Effect size of Atrial fibrillation on each individual outcome was quantified by crude odds ratios (OR), followed by adjusted OR after accounting for Euro SCORE (Table 3). Subgroup analysis of the type of operation and the outcome variables in relation to presence of atrial fibrillation is depicted in Table 4. OR, 95% confidence intervals (CI) as well as exact *P*-values are reported. Statistical analysis was performed using SPSS version 17 (SPSS, Chicago, IL, USA). The *P* values were considered statistically significant when <0.05.

## 3. Results

Our cohort (*n* = 3777) consisted of 934 (25%) females and 2843 (75%) males. Age ranged from 19 to 89 years old with a mean (±1 S.D.) of 63.9 (±10.1) years old. In-hospital mortality overall was 3.3% (124 patients). The incidence of preoperative A Fib in our cohort was 11% (413 patients) while 89% of patients were in normal sinus rhythm preoperatively (*n* = 3364). Preoperative factors and patient characteristics among the two groups are summarized in Table 1. As shown in this table, patients with preoperative A Fib were on average 4.5 years older, more females, higher Euro SCORE, with higher New York heart association (NYHA) grades, but lower Canadian cardiovascular society (CCS) grades. The incidence of congestive heart failure, renal failure, chronic obstructive pulmonary disease, and cerebrovascular accidents were also higher in the A Fib group. A Fib patients had higher incidence of valve surgery or combination of CABG and valve surgery than sinus rhythm patients. There was no significant difference between the two groups in relation to diabetes, hypertension, peripheral vascular disease, extracardiac arteriopathy, preoperative intraaortic balloon pump (IABP) requirement, and operative priority. 

The full spectrum of postoperative complications and in-hospital mortality among the two groups is summarized in Table 2. As shown, mortality was significantly higher in A Fib group (9% versus 3%, *P* < 0.001). In addition, the lengths of both hospital and ICU stay were significantly higher in the A Fib group. All types of postoperative complications including low cardiac output state, pulmonary complications, renal failure, infective complications, gastrointestinal complications, and reexploration were significantly higher in A Fib patients. Neurological complications were the only exception where no significant increase was noted.

By using logistic regression analysis (Table 3), crude and adjusted odds ratios (OR) were calculated. By adjusting for potential confounding variable such as Euro SCORE, we found that preoperative atrial fibrillation is significantly associated with in-hospital mortality (OR 1.7), low cardiac output (OR1.3), prolonged ventilation (OR 1.4), infective complications (OR 1.5), gastrointestinal complications (OR 2.0), and ICU readmission (OR 1.6) compared to normal sinus rhythm patients. Subgroup analysis of the type of surgical procedure and magnitude of the adverse influence of atrial fibrillation on each outcome variable is shown in Table 4.

## 4. Discussion

A Fib remains the most common arrhythmia seen in cardiac surgery both preoperatively and postoperatively. Various studies have examined the effect of postoperative A Fib on patients' outcome following cardiac surgery. However, only limited studies have examined the effect of preoperative A Fib on patients' outcome following cardiac surgery [4–8]. As summarized in Table 5, all these studies have inherent limitations such as type of surgery performed, the number of patients enrolled, and the type of morbidity examined. To date, our study remains the only study which comprehensively examined all types of postoperative complications in A Fib patients undergoing cardiac surgery. This is important as factors such as advancing age is a risk factor for A Fib, and cardiac surgery patients are increasingly referred at older age where this problem is most common. An understanding of the pattern of such complications and the effect of A Fib on outcome following surgery can lead to potentially preventative strategies to decrease the burden of these complications such as the use of concomitant Cox-MAZE procedure.

As shown in Tables 3 and 4, there was an increase in the rate of in-hospital mortality among A Fib patients compared to those in sinus rhythm. This effect persisted after adjusting for confounding variable such as Euro SCORE. In addition, low output state, gastrointestinal complications, and ICU readmission were also significantly increased in A Fib patients. Various reasons can potentially explain these observed effects. Preoperative atrial fibrillation can lead to low cardiac output postoperatively, hence the requirement of both rate control and inotropic support which in itself is arrhythmogenic. Our finding of low output state in preoperative A Fib patients is supported by previous observations [7, Table 5]. The effect of preoperative A Fib on gastrointestinal complications is a direct result of both low output state and embolic nature of such cases. These can lead to bowel ischemic or unstable gastrointestinal haemorrhages from low perfusion state [9]. Data on short-term mortality in A Fib patients are rather conflicting (Table 5). Some have showed increase in hospital mortality [7] while others did not. The main reason for the conflicting results stem from the number of patients analysed in these studies. Larger series showed significant association with hospital mortality while smaller series did not. This rise in mortality could be related to the complications observed in A Fib patients such as low output state and gastrointestinal complications and prolonged hospitalization or could be related to other factors perpetuated by A Fib itself.

The use of Cox-Maze procedure as a surgical strategy in dealing with preoperative A Fib patients is increasingly used. Data supporting the use of such procedure showed success rate of over 90% with low postoperative risk [10]. In addition, it also showed significantly reduced cost compared to medical therapy alone [10]. Our data show the increased risks of mortality and major morbidities may lend support to the routine inclusion of such procedure in A Fib patients undergoing cardiac surgery. Although, this has to be studied in dedicated studies.

## 5. Limitation of Study

There are few inherent limitations in our study. Firstly, our study is a retrospective study and as such we can only report an association rather than causality which could only be established by a randomized controlled trial. Secondly, no long-term followup is available for our patients that were included in this series. Thirdly, our data base does not record type of atrial fibrillation preoperatively (i.e., whether paroxysmal or chronic) which may or may not have different impact on outcomes observed. However, within these limitations, we believe our work does raise important observation that will stimulate more research into some preventative strategies when dealing with patients with preoperative atrial fibrillation.

## 6. Conclusion

As shown by our work and supported by other observations from literature, preoperative atrial fibrillation does increase the risk of mortality and major complications following cardiac surgery. Surgical strategies such as Cox-Maze procedure may be beneficial in these patients. 

## Figures and Tables

**Table 1:** Preoperative variables among sinus rhythm and atrial fibrillation patients (*n* = 3777).

Variable	Atrial fibrillation (*n* = 413)	Normal rhythm (*n* = 3364)	*P*-value
Age (years)			
Mean ± SD	67.9 ± 9.4	63.4 ± 10.1	<0.001
Gender			
Female	126 (31%)	808 (24%)	0.004
Euro SCORE (additive)			
Mean ± SD	6.3 ± 3.2	3.8 ± 2.8	<0.001
Angina class (CCS)			
CCS 3-4	179 (43%)	1777 (53%)	<0.001
NYHA score			
Moderate-severe	277 (67%)	1344 (40%)	<0.001
Congestive cardiac failure	203 (49%)	469 (14%)	<0.001
Number of previous MI			
None	294 (71%)	2032 (60%)	<0.001
One	92 (22%)	1103 (33%)	
Two or more	27 (7%)	229 (7%)	
Interval of MI to surgery			
None	294 (71%)	2032 (60%)	<0.001
<90 days	62 (15%)	591 (18%)	
>90 days	57 (14%)	741 (22%)	
Diabetes mellitus	76 (18%)	601 (18%)	0.789
Hypercholeterolemia	210 (51%)	2359 (70%)	<0.001
Hypertension	228 (55%)	1816 (54%)	0.638
Smoking status			
Current smoker	42 (10%)	532 (16%)	0.007
Former smoker	226 (55%)	1787 (53%)	
None smoker	145 (35%)	1045 (31%)	
Renal failure	51 (12%)	172 (5%)	<0.001
Chronic obstructive pulmonary disease	65 (16%)	295 (9%)	<0.001
Cerebrovascular accident	36 (9%)	175 (5%)	0.003
Peripheral vascular disease	63 (15%)	541 (16%)	0.665
Extracardiac arteriopathy	24 (6%)	189 (6%)	0.873
Extent coronary artery disease			
Single/double vessel	101 (24%)	770 (23%)	
Triple vessel	159 (39%)	2140 (64%)	<0.001
None	153 (37%)	454 (13%)	
Left main stem disease	67 (16%)	823 (24%)	<0.001
Ejection fraction			
<50%	209 (51%)	1198 (36%)	<0.001
>50%	204 (49%)	2166 (64%)	
Intra-aortic balloon pump	7 (2%)	34 (1%)	0.206
Operative priority			
Elective/urgent	394 (95%)	3252 (97%)	
Emergency/salvage	19 (5%)	112 (3%)	0.183
Cardiac procedure			
CABG	140 (34%)	2624 (78%)	
CABG + valve	102 (25%)	263 (8%)	<0.001
Valve	171 (41%)	477 (14%)	
BMI (kg/m^2^)			
Mean ± SD	26.9 ± 4.6	27.7 ± 4.8	0.003
Cardiopulmonary bypass time (min)			
Mean ± SD	121.0 ± 45.7	98.5 ± 40.8	<0.001
Cross-clamp time (min)			
Mean ± SD	79.0 ± 32.7	58.6 ± 24.9	<0.001

BMI: body mass index.

CABG: coronary artery bypass graft.

CCS: canadian cardiovascular society.

MI: myocardial infarction.

NYHA: New York heart association.

**Table 2:** Postoperative outcome in atrial fibrillation patients undergoing cardiac surgery compared to sinus rhythm patients (*n* = 3777).

Outcome	Atrial fibrillation (*n* = 413)	Normal rhythm (*n* = 3364)	*P*-value
Low cardiac output requiring Inotropes ± IABP	241 (58%)	1308 (39%)	<0.001
Reoperation	34 (8%)	182 (5%)	0.020
Ventilation time			
<24 hours	348 (84%)	3161 (94%)	<0.001
>24 hours	65 (16%)	203 (6%)	
Pulmonary complication	97 (23%)	640 (19%)	0.031
Neurological complications	37 (9%)	228 (7%)	0.101
Renal failure			
Dialysis/nondialysis	53 (13%)	208 (6%)	<0.001
No	360 (87%)	3156 (94%)	
Infective complications	60 (15%)	253 (8%)	<0.001
Gastrointestinal complications	13 (3%)	39 (1%)	<0.001
ICU stay (days)			
Mean ± SD	3.4 ± 7.4	1.9 ± 4.3	<0.001
ICU readmission	29 (7%)	99 (3%)	<0.001
Length of hospital stay (days)			
Mean ± SD	12.7 ± 17.4	8.7 ± 11.4	<0.001
Blood transfusion	231 (56%)	1486 (44%)	<0.001
Status			
Alive	375 (91%)	3278 (97%)	<0.001
Dead	38 (9%)	86 (3%)	

ICU: intensive care unit.

IABP: intraaortic balloon pump.

**Table 3:** Logistic regression analysis for atrial fibrillation and outcome (unadjusted and Euro SCORE adjusted) (*n* = 3777).

	Unadjusted			Euro SCORE Adjusted		
	OR	95% C.I	*P*-value	OR	95% C.I	*P*-value
Death	3.9	(2.6–5.7)	<0.001	1.7	(1.1–2.7)	0.013
Low Cardiac	2.2	(1.8–2.7)	<0.001	1.3	(1.03–1.6)	0.026
Reoperation	1.6	(1.1–2.3)	0.020	1.1	(0.8–1.7)	0.501
Ventilation	3.0	(2.2–3.9)	<0.001	1.4	(1.0–1.96)	0.048
Pulmonary	1.3	(1.0–1.7)	0.030	0.96	(0.7–1.2)	0.734
Neurological	1.4	(0.9–2.0)	0.100	0.9	(0.6–1.3)	0.507
Renal	2.2	(1.6–3.1)	<0.001	1.3	(0.9–1.8)	0.197
Infective	2.1	(1.5–2.8)	<0.001	1.5	(1.1–2.1)	0.010
GI^∗^	2.8	(1.5–2.2)	0.002	2.0	(1.0–3.9)	0.044
ICU	2.5	(1.6–3.8)	<0.001	1.6	(1.0–2.5)	0.047
Blood transfusion	1.6	(1.3–1.8)	<0.001	0.8	(0.6–0.99)	0.043

^∗^GI: gastrointestinal complications.

ICU: intensive care unit readmission.

**Table 4:** Subgroup analysis of the type of surgery and magnitude of adverse influence of atrial fibrillation on each outcome variable (*n* = 3777).

		CABG			CABG + valve			Isolated valve	
	OR	95% C.I	*P*-value	OR	95% C.I	*P*-value	OR	95% C.I	*P*-value
Death	2.0	0.98–4.2	0.056	2.5	1.0–5.9	0.045	1.5	0.7–3.5	0.319
Low cardiac	0.8	0.5–1.1	0.206	1.9	1.1–3.2	0.021	1.99	1.4–2.9	<0.001
Reoperation	1.3	0.6–2.7	0.503	1.1	0.5–2.2	0.834	0.7	0.3–1.4	0.299
Ventilation	0.97	0.5–1.9	0.926	1.6	0.9–2.9	0.135	1.4	0.8–2.4	0.284
Pulmonary	1.2	0.8–1.8	0.425	1.2	0.7–2.0	0.433	0.7	0.4–1.2	0.190
Neurological	0.7	0.3–1.4	0.337	0.9	0.4–1.9	0.815	0.97	0.5–1.8	0.927
Renal	0.9	0.5–1.8	0.806	2.3	1.2–4.2	0.011	0.99	0.5–1.8	0.973
Infective	1.1	0.6–1.9	0.769	2.7	1.5–4.8	0.001	1.7	0.9–3.4	0.132
GI^∗^	2.1	0.7–6.4	0.177	2.7	0.9–7.8	0.075	0.9	0.1–5.3	0.889
ICU^∗∗^	1.4	0.6–3.0	0.428	2.1	0.9–4.9	0.099	1.6	0.7–3.7	0.286
Blood	0.8	0.5–1.1	0.157	0.8	0.5–1.3	0.344	0.8	0.6–1.2	0.402

CABG: coronary artery bypass graft.

^∗^GI: gastrointestinal complications.

^∗∗^ICU: intensive care unit readmission.

**Table 5:** Summary of previously published studies examining the effect of preoperative atrial fibrillation (A Fib) on outcome following cardiac surgery.

First author/year	Type of study/number	Main findings	limitations/comment
Ngaage et al. [4], 2006	Retrospective *n* = 381	(1) A Fib patients had significantly lower survival at 1, 5, and 7 years than sinus rhythm patients. (2) A Fib patients had higher incidences of stroke, congestive heart failure and rhythm-related intervention on followup. (3) A Fib was an independent predictor of late adverse cardiac and cerebrovascular events but not late death. (4) A trend towards increase in-hospital mortality among A Fib patients.	(1) Patients were matched for age, gender, and ejection fraction. (2) Only included aortic valve replacements.

Rogers et al. [5], 2006	Retrospective *n* = 5092	(1) A Fib occurred in 3.4% of patients undergoing isolated elective CABG. (2) No difference in hospital mortality between sinus rhythm and A Fib patients. (3) A Fib patients had longer hospital stay and higher requirement of intraaortic balloon pump. (4) A Fib patients had 49% higher risk of death after 5 years of surgery than sinus rhythm patients.	(1) Only elective CABG (on-pump and off-pump). (2) Limited morbidities were examined.

Ngaage et al. [6], 2007	Retrospective *n* = 526 (matched analysis)	(1) A Fib occurred in 8.3% of patients undergoing isolated CABG. (2) Operative mortality was similar between sinus rhythm and A Fib patients. (3) A Fib patients had longer hospitalization. (4) A Fib patients had more late hospital admission (median followup 6.7 years). (5) A Fib patients had 40% higher late mortality risk from all causes compared to sinus rhythm patients with more MACE in A Fib patients. (6) A Fib patients had 2.1 relative risk of pacemaker insertion than sinus rhythm patients.	(1) Only on-pump CABG included. (2) Small sample size overall. (3) Only examined MACE, stroke, mortality and hospital stay.

Banach et al. [7], 2008	Retrospective *n* = 3000	(1) A Fib patients had lower survival at 6, 12, and 36 months post CABG. (2) A Fib was an independent risk factor for in-hospital death. (3) A Fib patients had 20% lower survival difference than sinus rhythm patients. (4) A Fib was associated with prolonged ventilation, low output state and prolonged hospital stay and ICU stay	Only CABG patients were included. The risks of other major complications were not evaluated.

Fukahara et al. [8], 2010	Retrospective *n* = 513	(1) Preoperative A Fib occurred in 5.1% of patients. (2) No difference in operative mortality between A Fib and sinus rhythm patients. (3) A Fib patients had significantly lower survival at 5 years than sinus rhythm patients (70% versus 87%). (4) Freedom from cerebral complications was significantly decreased in A Fib patients. (5) No difference in cardiac mortality or MACE at 5 years. (6) A Fib was a significant adverse predictor of survival and independent predictor of late cerebral infarction.	(1) Only off-pump CABG included. (2) Transient ischemic attacks and cerebral haemorrhages were not counted as cerebrovascular events. (3) Sample size was small to detect early association between A Fib and postoperative mortality or morbidity. (4) Postoperative morbidities were not examined.

ICU: intensive care unit.

MACE: major adverse cardiac events.

## References

[1] Ryder KM, Benjamin EJ (1999). Epidemiology and significance of atrial fibrillation. *American Journal of Cardiology*.

[2] Athanasiou T, Aziz O, Mangoush O (2004). Do off-pump techniques reduce the incidence of postoperative atrial fibrillation in elderly patients undergoing coronary artery bypass grafting?. *Annals of Thoracic Surgery*.

[3] Gammie JS, Haddad M, Milford-Beland S (2008). Atrial fibrillation correction surgery: lessons from the society of thoracic surgeons national cardiac database. *Annals of Thoracic Surgery*.

[4] Ngaage DL, Schaff HV, Barnes SA (2006). Prognostic implications of preoperative atrial fibrillation in patients undergoing aortic valve replacement: is there an argument for concomitant Arrhythmia Surgery?. *Annals of Thoracic Surgery*.

[5] Rogers CA, Angelini GD, Culliford LA, Capoun R, Ascione R (2006). Coronary surgery in patients with preexisting chronic atrial fibrillation: early and midterm clinical outcome. *Annals of Thoracic Surgery*.

[6] Ngaage DL, Schaff HV, Mullany CJ (2007). Does preoperative atrial fibrillation influence early and late outcomes of coronary artery bypass grafting?. *Journal of Thoracic and Cardiovascular Surgery*.

[7] Banach M, Goch A, Misztal M (2008). Relation between postoperative mortality and atrial fibrillation before surgical revascularization—3-Year follow-up. *Thoracic and Cardiovascular Surgeon*.

[8] Fukahara K, Kotoh K, Doi T, Misaki T, Sumi S (2010). Impact of preoperative atrial fibrillation on the late outcome of off-pump coronary artery bypass surgery. *European Journal of Cardio-thoracic Surgery*.

[9] Chaudhuri N, James J, Sheikh A, Grayson AD, Fabri BM (2006). Intestinal ischaemia following cardiac surgery: a multivariate risk model. *European Journal of Cardio-thoracic Surgery*.

[10] Poynter JA, Beckman DJ, Abarbanell AM (2010). Surgical treatment of atrial fibrillation: the time is now. *Annals of Thoracic Surgery*.

